# How AlphaFold2 shaped the structural coverage of the human transmembrane proteome

**DOI:** 10.1038/s41598-023-47204-7

**Published:** 2023-11-20

**Authors:** Márton A. Jambrich, Gabor E. Tusnady, Laszlo Dobson

**Affiliations:** 1 Protein Bioinformatics Research Group, Institute of Enzymology, Research Centre for Natural Sciences, Magyar Tudósok Körútja 2, Budapest, 1117 Hungary; 2 Department of Bioinformatics, Semmelweis University, Tűzoltó u. 7, Budapest, 1094 Hungary; 3 Structural and Computational Biology Unit, European Molecular Biology Laboratory, Meyerhofstraße 1, 69117 Heidelberg, Germany

**Keywords:** Protein structure predictions, Molecular modelling

## Abstract

AlphaFold2 (AF2) provides a 3D structure for every known or predicted protein, opening up new prospects for virtually every field in structural biology. However, working with transmembrane protein molecules pose a notorious challenge for scientists, resulting in a limited number of experimentally determined structures. Consequently, algorithms trained on this finite training set also face difficulties. To address this issue, we recently launched the TmAlphaFold database, where predicted AlphaFold2 structures are embedded into the membrane plane and a quality assessment (plausibility of the membrane-embedded structure) is provided for each prediction using geometrical evaluation. In this paper, we analyze how AF2 has improved the structural coverage of membrane proteins compared to earlier years when only experimental structures were available, and high-throughput structure prediction was greatly limited. We also evaluate how AF2 can be used to search for (distant) homologs in highly diverse protein families. By combining quality assessment and homology search, we can pinpoint protein families where AF2 accuracy is still limited, and experimental structure determination would be desirable.

## Introduction

Transmembrane (TM) proteins are gatekeepers that regulate the movement of nutrients, metabolites and drugs in and out of cells while also sensing, transmitting or amplifying signals in neurotransmission and chemical perception. After the accomplishment of the Human Genome Project, it was determined that around 20–30% of genes in the human genome encode transmembrane proteins (TMPs), which means that there are approximately 5–7 thousand TMPs in the human proteome^1^. The lipid bilayer surrounding the TMPs exposes them to distinct phases (both hydrophobic and hydrophilic), which require a multi-step folding process for proper embedding^2^. Despite the crucial role that TMPs play in cellular processes, their structural characterization lags far behind that of globular proteins due to their naturally dual environment^3^, which makes their recombinant expression, purification and crystallization difficult^4^.

These experimental challenges have increased the demand for various computational approaches, ranging from topology prediction^5^ (which determines the exact location of TM segments and the orientation of connecting loops relative to the membrane, e.g., cytosolic, extracellular), to the 3D prediction of TMPs^6^. The latter has become an important field, with approaches borrowed from the prediction of globular proteins and extended with special conditions arising from the topology during contact map definition^7^. Despite enormous efforts, the accuracy of these topology and 3D predictions is limited, because the training set for fine-tuning these methods is always limited by the number of experimentally solved structures. In the 2000s numerous structural genomic projects began to pave the way for more inclusive membrane protein structure determination^8,9^, with definite, but limited success. The situation has only improved over the last 10 years, as cryo-electron microscopy (cryo-EM) has revealed hundreds of new TMP structures.

However the breakthrough for membrane protein structures seems to have come from a computational tool: AlphaFold2^10^ (AF2), developed by the team at DeepMind, has revolutionized the field of structural biology by greatly improving the speed and accuracy of protein structure prediction. The software uses deep learning algorithms and a vast amount of protein sequence and structural data to predict the 3D structure of a protein based solely on its amino acid sequence. It was quickly realized that AF2 could be used in many ways for structural biology research, including experimental mode ling, prediction of intrinsically disordered regions, or assessment of disease-causing mutations^11^. However, the question remained as to how AF2 performs in the field of membrane proteins where the training data it could use was still very limited.

We have recently launched the TmAlphaFold database^12^, which adds the possible localization of the lipid bilayer relative to the AF2-predicted helical TMP structures and evaluates the quality of each prediction. In this study, we analyze the human TM proteome to identify issues, where we encountered limitations of the AF2 structures. We made an automatic quality assessment of the state of the human (predicted) TM proteome, performed sequence- and structure-based clustering of TMPs, and then highlighted proteins or protein families where there is still room for improvement. Our goal was to identify the source of potential errors and/or protein families where AI-based prediction is still not possible to a satisfactory degree.

## Results

### TmAlphaFold database is a good starting point for discriminating between high and low quality structures

The TmAlphaFold database^12^ not only provides a possible localization of the lipid bilayer in the predicted structures but also evaluates the quality of the predicted membrane-embedded structures. We constructed a non-redundant human TMP benchmark set consisting of proteins with (I) solved 3D structures3 (3D_set) and (II) high quality topography predictions from the Human Transmembrane Proteome (HTP) (topography_set) database1 (the position of the TM helices in the amino acid sequence—not to mistake with topology prediction, where cytosolic/extracellular localization of the loops are also provided).

We classify an AlphaFold2 structure as high quality from a “membrane point of view”, when in the TMAlphaFold database the membrane plane is defined correctly. To distinguish the correct from erroneous structures, we made a topography comparison using the following procedure: Proteins in the topography_set already had a defined topography. For proteins in the 3D_set structures, we used the lipid bilayer position from the PDBTM database, and then made a 2D projection to define their topography. These topographies were then used as a reference. Next, we defined the topography of AF2 structures after obtaining the position of the membrane bilayer from the TmAlphaFold database and made the same 2D projection as we did for experimental structures. AF2 modeled structures were considered correct, when their topographies agreed with the reference topography (the number of TM segments was equal and all helices were covered with at least 5 amino acids).

**Figure 1:**
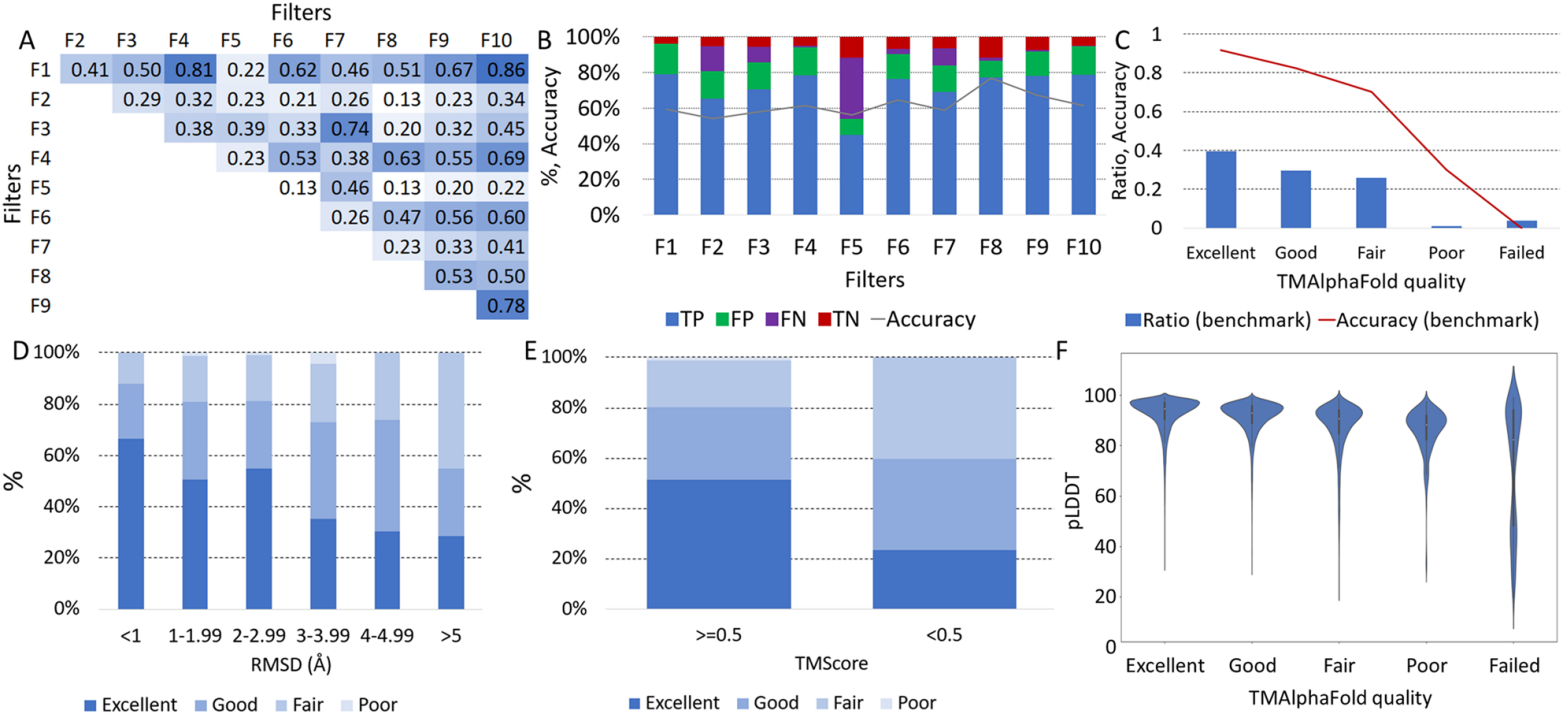
Evaluation of transmembrane AlphaFold2 structures: (**A**) Correlation between different filters used by TMAlphaFold on the 3D_set/topography_set (F1: DetectingMembranePlane, F2: Signal, F3: FullStructure, F4: ShortHelix, F5: Masked, F6: MissingTmpart, F7: Domain, F8: OverpredictCctop, F9: UnderpredictCctop, F10: MembranePlaneCctop). Darker shade of blue means higher correlation. For an explanation of these filters, see the main text; (**B**) Performance of different filters on the 3D_set/topography_set; (**C**) Relative number of proteins and the measured accuracy (by checking topography) at different quality levels on the 3D_set/topography_set; (**D**) Distribution of TMAlphaFold quality levels at different RMSD distances (in Å) between the experimental and model template in the 3D_set; (**E**) Distribution of TMAlphaFold quality levels at different TM-Score groups in the 3D_set; (**F**) Distribution of pLDDT values in the transmembrane regions at different TMAlphaFold quality levels on the 3D_set.

TmAlphaFold uses filters for quality assessment. These filters flag errors in the structure, for example if a globular domain is embedded in the membrane, there is a conflict with the topography prediction, when intramembrane regions are folded outside the bilayer plane, and more (see TMalphaFold web page for details). First, we checked whether the different filters capture unique errors to rule out that they all detect the same problems but with a different approach. Using the 3d_set/topography_set, we calculated the correlation between the filters and found that most of the filters were not correlating (Fig. 1A).

We also wanted to find out the performance of different filters (Fig. 1B) considering the (in)correct structures defined in the 3D_set/topography_set. Most filters are typically more sensitive and less specific, allowing several incorrect structures to pass the filter (high amount of false positives). In addition, F2 and F5 are also less sensitive, because these errors are more easy to fix: both signal peptides and low pLDDT regions are quite trivial to mask out. These results indicate that the filter alone cannot be used to determine the quality of the AF2 structures. Filters can detect different errors, but even if a protein fails many filters sometimes the membrane can be defined, or vice versa, a protein passing all filters can be still problematic.

Next we used the 5 quality levels used in the TmAlphaFold database, defined by the sum of filters the protein passes, categorizing structures from failed to excellent. Excellent quality proteins are indeed topographically correct in 97% of the cases, while the number of errors increases in lower quality categories (Fig. 1C). It is also visible that, considering the 3D_ set/topography_set, 40% of the proteins belong to the Excellent category, and less than 10% of the proteins have Poor or Failed categories.

For proteins in the 3D_set we also calculated the Root Mean Square Deviation (RMSD) and TM-Score using TM- align^13^. When the experimental and predicted structures have a low RMSD (< 1 Å), almost 90% of the proteins have Good or Excellent quality, while the higher the RMSD, the lower the proportion of Good and Excellent structures (Fig. 1D). The same is true for the TM-Score (Fig. 1E), where above the 0.5 threshold, 80% of the proteins have Good or Excellent quality, while below the 0.5 threshold, only 59% are of Good or Excellent quality.

AF2 also provides a model self-assessment in terms of a confidence value for each predicted residue, called the predicted Local Distance Difference Test (pLDDT): the distribution of these values for TM regions shows that AF2 is more certain for higher quality predictions (Fig. 1F), as expected.

### How AlphaFold2 shaped the coverage of the human transmembrane proteome

**Figure 2:**
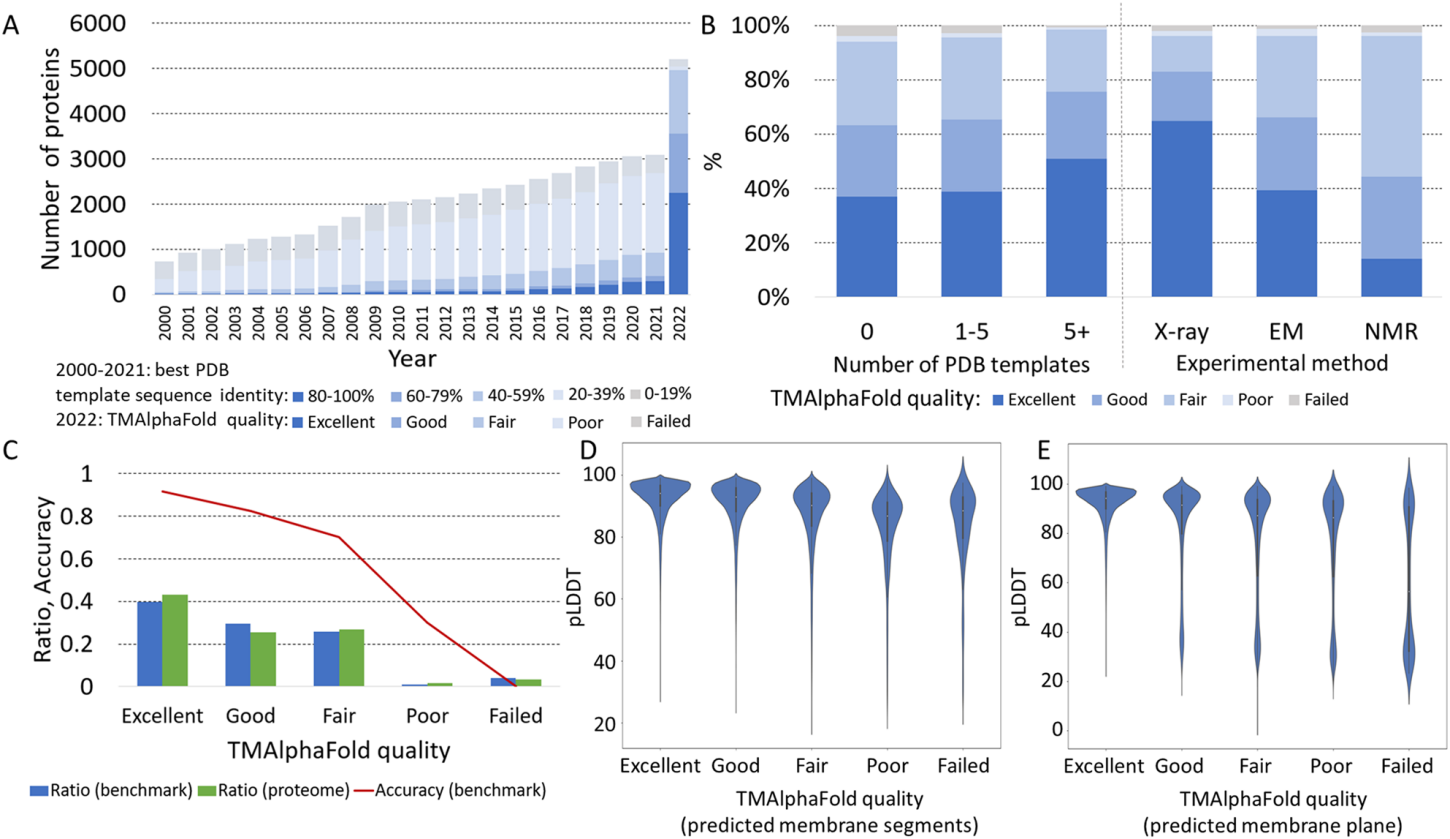
Coverage of the human transmembrane proteome: (**A**) Structural coverage of TM proteins (from 2000 to 2021) according to their sequence identity to PDB entries and TMAlphaFold (2022) structures based on their quality level; (**B**) Distribution of TMAlphaFold quality levels on proteins with and without homologous structures and also based on experimental technique by which the template structure has been determined; (**C**) Distribution the number of TMAlphaFold structures at different quality levels (3D_set/topography_set results are also displayed for comparison); (**D**) Distribution of pLDDT values in the HTP predicted transmembrane regions at different TMAlphaFold quality levels; (**E**) Distribution of pLDDT values in the TMAlphaFold detected transmembrane regions at different TMAlphaFold quality levels.

Next, we extended our analysis to the entire human TM proteome. First, we examined how the structural coverage (in terms of high-quality model availability) of the TM proteome changed after AF2 was released. For this task, we used HHBlits^14^ to search for PDBTM structures where the deposited structure covered all TM helices. For each year, we only incorporated PDB^15^ structures that were available at that time. We categorized the hits based on sequence identity and created a graph to visualize the data (see Fig. 2A). In the early 2000s, the number of experimentally determined structures with high sequence identity (> 80%) was less than 100. Even when considering very distant homologs (< 20%), about 20% of the TM proteome had a recognizable paralog. Notably, this also marked the upper limit of proteins for which 3D structure prediction could be performed using homology-recognition or threading approaches. As more research groups gained access to cryo-EM, the number of close (and distant) homologs increased, showing a promising trend. However, it would have taken decades to cover the entire TM proteome with high-quality templates. If we add AF2 structures to this graph using the TmAlphaFold quality score, the impact of AF2 becomes readily apparent, with more than half of the TM proteome falling into the Good and Excellent categories (see Fig. 2A).

Although AF2 performs quite well, it is a supervised method, and this becomes evident when we check the quality of proteins for which no homologous protein was available in the PDB and for which 1–5 or more template structures were already solved (see Fig. 2B). The number of excellent structures is slightly higher when there are a few homologs, but AF2's performance is convincing when there are at least five homologs. AF2 has been trained on X-ray structures and therefore performs better on proteins with available X-ray templates than on proteins with only NMR or cryo-EM homologs in the PDB (see Fig. 2B).

The distribution of proteins in different quality categories on the 3D_set/topography_ set and the whole proteome is almost identical; therefore, we can assume that about 40% of the whole TM proteome has 97% accuracy (considering topography), and about 90% of the TM proteome has a 70% accuracy (see Fig. 2C).

We also calculated the distribution of pLDDT values on (I) predicted/projected TM regions (see Fig. 2D) and (II) within the modeled lipid bilayer according to TMAlphaFold (see Fig. 2E). In both cases, the higher the structure quality category, the higher the mean pLDDT value in the membrane regions. In the case of the modeled lipid bilayer, the distribution is bimodal on all quality levels except Excellent, probably reflecting the flexible protein segments that violate the membrane plane. Since the lipid bilayer was not defined for AF2 training, disordered regions often cross the bilayer, resulting in incorrectly located domains.

### High quality AlphaFold2 structures provide a solid base for fast and accurate searching and clustering of structural models

Searching for distant homologs and clustering protein sets are commonly used to associate annotations to protein IDs or to generate benchmark sets. In this section, we aimed to figure out if Foldseek^16^ clustering based on AlphaFold2 transmembrane structures performs better than traditional (sequence based) homology searching tools. Accordingly, we collected protein sets from several databases, including CATH^17^, UniProt^18^, Membranome^19^, and the Transporter Classification Database^20^. We then manually discarded proteins or merged groups to create protein families with the same architecture (e.g. number of TM segments) that we named homology_set.

We first searched for homologs using HHBlits, BLAST^21^, and Foldseek (using the full AF2 structures and also using parts of the structure that are within the TMAlphaFold predicted bilayer + -5 Ångström). In all cases, we used two approaches: one where all hits were accepted, and another where only hits with the same number of TM segments were accepted (TMs covered) compared to the reference query. Notably, for homology-modeling purposes, only the latter once can be accepted, as in other cases hits might only cover a globular fragment of the membrane protein. Supplementary Fig. 1 helps to better understand how homology pairs were defined in the homology_set and how hits were selected. For sequence-based approaches, we used topographies from the HTP database, while for AF2 structures, we used topographies derived from TMAlphaFold structures. The sequence and structure-based searches yielded similar results (Table 1): in both cases, the best achievable sensitivity was around 0.87, and the best specificity was close to 1. Considering more universal metrics that take into account both positive and negative cases (Matthew’s Correlation Coefficient: MCC and Balanced Accuracy: BACC), HHBlits and Foldseek provided the best results.

**Table 1:** Homology searching results using different approaches.

Approach	TP	FP	FN	TN	Sens	Spec	BACC	MCC	AUC/prob	AUC/seqID	AUC/TM-Score
HHBlits all	24,456	4227	3424	73,463	0.877	0.946	0.911	0.815	0.915	0.932	×
HHBlits Tms covered	23,315	3137	4565	74,553	0.836	0.960	0.898	0.810	0.894	0.905	×
BLAST all	21,979	597	5901	77,093	0.788	0.992	0.890	**0.837**	0.893	0.893	×
BLAST Tms covered	20,316	73	7564	77,617	0.729	**0.999**	0.894	0.813	0.859	0.859	×
Foldseek all	24,466	3528	3414	74,162	0.878	0.955	0.916	0.831	0.932	×	0.931
Foldseek Tms covered	20,715	992	7165	76,698	0.743	0.987	0.865	0.814	0.797	×	0.891
Foldseek_mem all	21,326	294	6554	77,396	0.765	0.996	0.881	0.831	0.882	×	0.881
Foldseek_mem Tms covered	16,884	165	10,996	77,525	0.606	0.998	0.802	0.723	0.802	×	0.802
NNSearch	25,218	4168	2662	73,522	**0.905**	0.946	**0.925**	**0.837**	**0.985**	×	×

*TP* True positive, *FP* False positive, *FN* False negative, *TN* True negative, *Sens* Sensitivity, *Spec* Specificity, *BACC* Balanced Accuracy, *MCC* Matthew’s Correlation Coefficient. *AUC* Area Under Curve (based on e-value for HHBlits, BLAST and Foldseek, and probability for NNSearch).

Highest values are in [bold].

**Figure 3:**
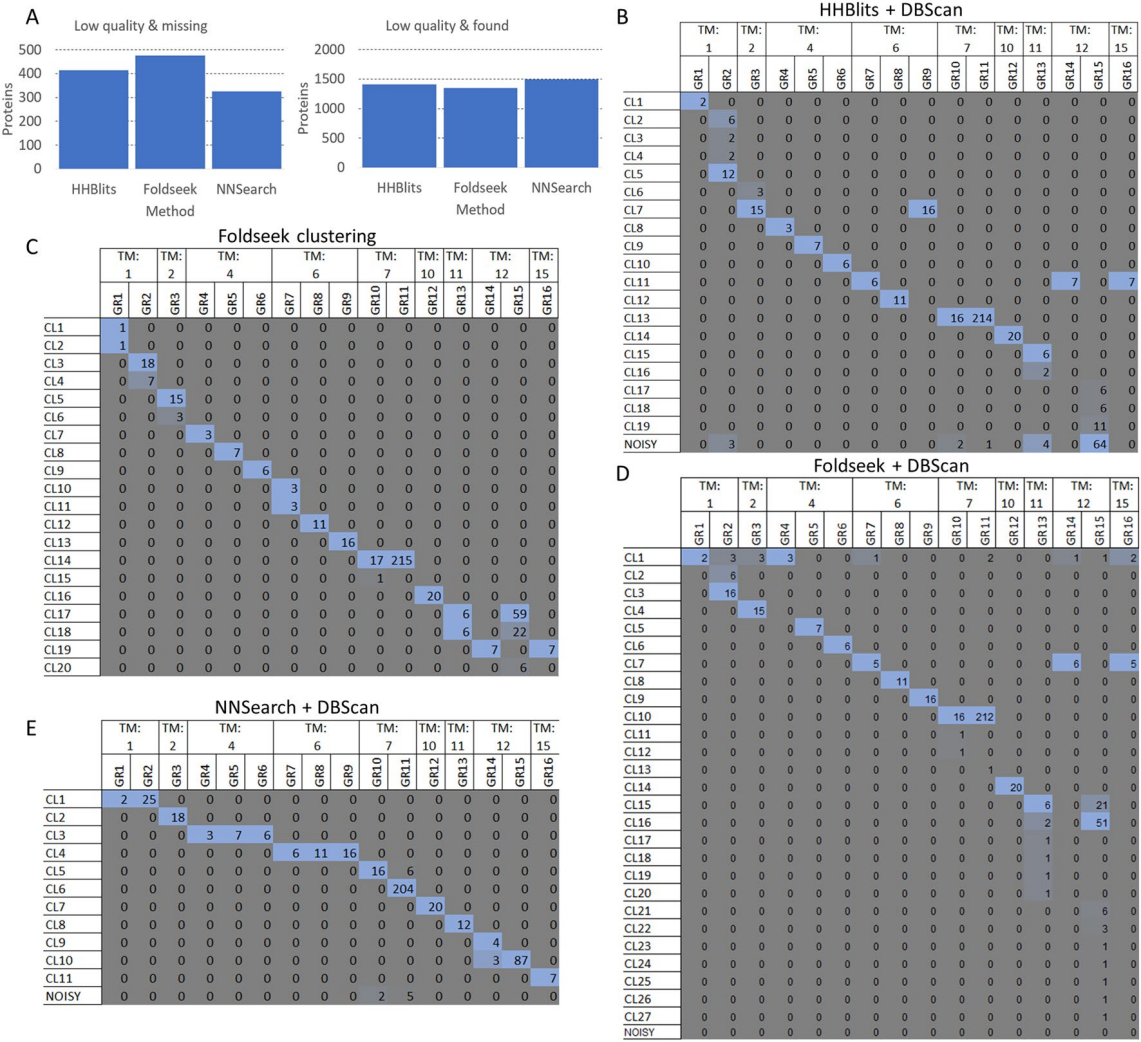
Sequence and structure based search and clustering results: (**A**) Left: Number of missing proteins (false negative) with “Fair” or worse quality using different approaches. Right: Number of found proteins (true positive) with “Fair” or worse quality using different approaches. (**B**) Number of proteins in different clusters categorized by protein families (GR1: Single helix bin, GR2: Integrin, GR3: KCN_3, GR4: Eic/Glu me. Domain, GR5: Neurotim ion-channel, GR6: CACN_2, GR7: ABC_2, GR8: Aquaporin, GR9: KCN_1, GR10: Rhodopsin, GR11: Olfactory, GR12 :ATP_1, GR13: SLC_1, GR14: ABC_1, GR15: SLC_2, GR16: ABC_3), using HHBlits and DBScan. (**C**) Number of proteins in different clusters categorized by protein families, using Foldseek clustering. (**D**) Number of proteins in different clusters categorized by protein families, using Foldseek and DBScan. (**E**) Number of proteins in different clusters categorized by protein families, using the Neural Network and DBScan.

Sequence-based and structural search approaches both have an advantage for different proteins and protein sets—for example, structural approaches are likely to fail if the structures provided are of poor quality. To overcome this problem we developed a simple neural network (NNSearch) using search results from all methods that were added as an input. To avoid possible overfitting, several neural networks were built, each time using a single protein family for testing, and all other protein families for training. The results of individual predictors were aggregated to predict homology (see Methods). NNSearch achieved the highest sensitivity, balanced accuracy, and MCC (Table 1). Looking at the number of missing pairs (i.e. false negatives) with “Fair” or worse quality, Foldseek struggles to find relationships in these cases compared to HHBlits (Fig. 3A). By combining the sequential and structural search modes, poor-quality structures have a lower impact on finding homologs. Similarly, the number of hits found (i.e. true positive) with “Fair” or worse quality is the lowest with Foldseek, and the highest with NNSearch (Fig. 3A).

Next, we used DBScan^22,23^ on HHBlits to cluster protein pairs into groups (Fig. 3B). To select the best clustering result from multiple settings, we calculated the Adjusted Rand Index (ARI) and the Adjusted Mutual Information (AMI) after iterating through all reasonable parameters. Not unexpectedly for deep homology search algorithms, several families that were separated by topography in the homology_ set ended up in the same cluster. However, this behaviour cannot be considered as a serious error, as architectures repeating the same domain(s) but different numbers of occurrences were found in these cases (e.g. ABC protein families).

We then used the built-in clustering approach of Foldseek (Fig. 3C) on the full structures: its performance was superior to sequential homology search and DBScan clustering. Some protein families were split into two groups. Merging of different groups occurred mainly for ABC proteins and GPCR family members. The results can be adjusted by adding topography filters that do not allow proteins with different topographies to be in the same cluster. We also performed DBScan clustering on pairs generated by Foldseek (Fig. 3D), which gave similar results to the built-in clustering method, except that it split the SLC_2 family into 4 clusters.

Finally, we performed DBScan clustering on the result of NNSearch (Fig. 3E). GPCR-s are by far the largest membrane protein families and this approach successfully separated olfactory receptors and rhodopsins, but other protein families with the same number of TM segments were clustered together.

DBScan clustering on the NNSearch results yielded the highest ARI (0.92) and AMI (0.90) scores, before HHBlits (ARI: 0.84; AMI: 0.82), Foldseek (ARI: 0.83; AMI: 0.79), while the FoldSeek clustering alone achieved 0.85 ARI and 0.85 AMI score.

### Ups and downs of AlphaFold2 prediction quality

We also looked for groups of proteins for which AF2 gave confusing results. For this task, we used NNSearch that is based on HHBlits, BLAST, and Foldseek and clustered the human proteome using DBScan. DBScan categorized over a thousand proteins into the noisy cluster, and there is also a suspiciously large group dominated by bitopic membrane proteins. Altogether there are 197 protein clusters. For further analysis, we converted the TMAlphaFold quality level to a scale of 1–5 (from failed to excellent).

There are 59 protein clusters with at least 4.5 mean quality, including 660 proteins. TmAlphaFold quality seems to be high for several transporter families: Solute carrier family 25/35 members, amino acid transporters and more (Table 2 shows these clusters when they have at least 20 members).

**Table 2:** Clusters with a high number of good quality structures.

Cluster number	Dominant protein family	Number of members	Mean quality	Number of TM segments
36	Tetraspanin	32	4.90625	4
5	Solute carrier family 25 member	20	4.9	6
4	Claudin	57	4.894737	4
1	Solute carrier family 35 member	26	4.884615	10
17	Monocarboxylate transporter	71	4.859155	12
100	Sodium- and chloride-dependent amino acid transporter	28	4.821429	12
37	Glucuronosyltransferase	21	4.809524	1
73	Various bitopic proteins	22	4.727273	1
32	Palmitoyltransferase	20	4.7	4
25	Various receptors	46	4.652174	4

There are also 62 protein clusters, where the mean quality is below 3.5, containing 353 proteins in total. 40 clusters (146 proteins) of these groups do not have any 4 (Good) or better quality members. Proteins belonging to these clusters belong to the most elusive part of the TM protein space, and further experimental/computational work on them is desired to better understand their structure and function. There are an additional 31 clusters and 1763 proteins with a mean quality below 4. Notably, all of these clusters have at least one representative member with 4 or better quality.

**Figure 4:**
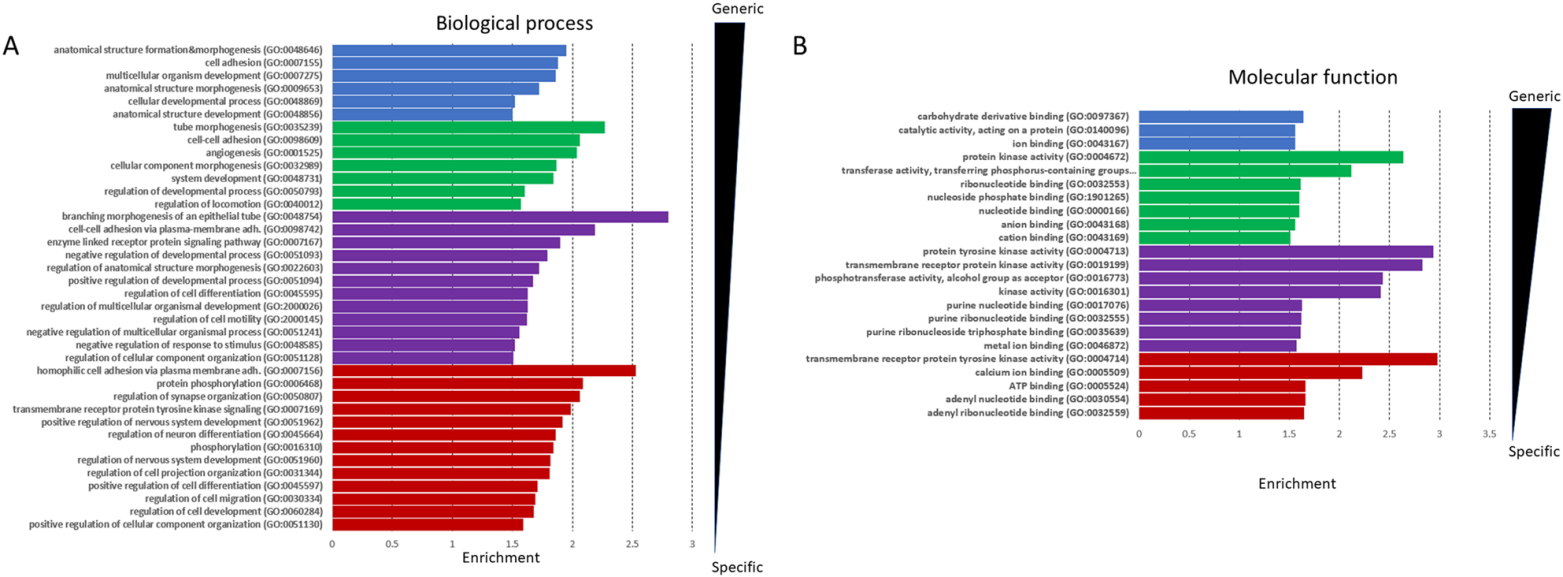
GeneOntology terms of bad quality structures: Highly significant terms are sorted based on their level in the GeneOntology tree (blue, green, purple, red: 2–5, respectively) and on fold enrichment. (**A**) Molecular Function (**B**) Biological process.

Additionally, there are several other protein families with generally low quality structures, such as members of the tumor necrosis factor receptor superfamily, some of the solute carrier families, glutamate receptors and others. Looking at the GeneOntology molecular function (Fig. 4A) analysis for Fair quality structures or below (using all human TM proteins as a background), terms related to kinase activity are enriched (proteins involved are likely to be disordered). For biological processes (Fig. 4B), cell adhesion (typically bitopic membrane proteins), protein phosphorylation (high disordered content) and synaptic/neuronal process related terms (large proteins with multiple modules) are enriched. For the cellular compartment cell surface (many bitopic proteins) and receptor complex (multimeric proteins) terms were enriched.

## Discussion

In this study, we evaluated the quality of alpha-helical membrane protein structures predicted by AlphaFold2. AF2 provided good and excellent quality structures for over 50% of the human TM proteome. Most of these proteins (or protein families) have a solved 3D structure and AF2 can rely on the template of the structure. Scientists often analyze their protein of interest or research focus, and in these highly studied areas, AF2 performs better (as these proteins have usually been studied for decades), for example in the case of ABC transporters^24^. Although AF2 has made a major contribution to the coverage of the human transmembrane proteome, there are still protein groups for which its performance seems to be poor. AF2 currently relies heavily on (X-ray structures in) the PDB, as the quality of predicted structures dropped dramatically when we considered proteins for which no template structure could be found. About 30% of the human proteome still lacks structures of convincing quality. One area where AF2 is limited is in multimeric proteins, where the correct fold is highly dependent on interactions. Although AF2 was quickly tailored by the scientific community to accept multiple protein sequences^25^, this is not yet reflected in the AlphaFold Protein Structure database^26^. Proteins with high levels of disorder also cause problems for AF2, for example despite the relatively high number of experimentally determined syndecan structures^27^, the quality of their models is poor due to the flexible regions of their ectodomains.

Homology searching and properly defining protein clusters is also a challenging goal for TM proteins. Multiple structure-based and guided methods have recently developed^28,29^ to improve homology searches, but all of these algorithms rely heavily on the quality of the input structures. AF2 structures provide a solid base for quick searching and clustering, sometimes outperforming traditional methods. Yet, in the case of TM proteins, we found that using AF2 structures for this task is only a good idea when the quality of the proteins is high (the TmAlphaFold database can be a good starting point) and sequence-based homology search methods still have room when the quality is lower.

In general, AF2 seems to have three main problems when predicting membrane proteins: (I) Flexible connecting loops often cross the bilayer and sometimes floating helices are also loosely placed into the membrane, especially in the case of bitopic TM proteins. (II) Membrane segments are predicted outside the lipid bilayer—most often followed by a flexible segment. (III) AF2 forces compact structures and folds non-TM alpha helices to the membrane plane. The following examples are not only clusters with multiple poor-quality structures but also represent the aforementioned errors.

**Figure 5:**
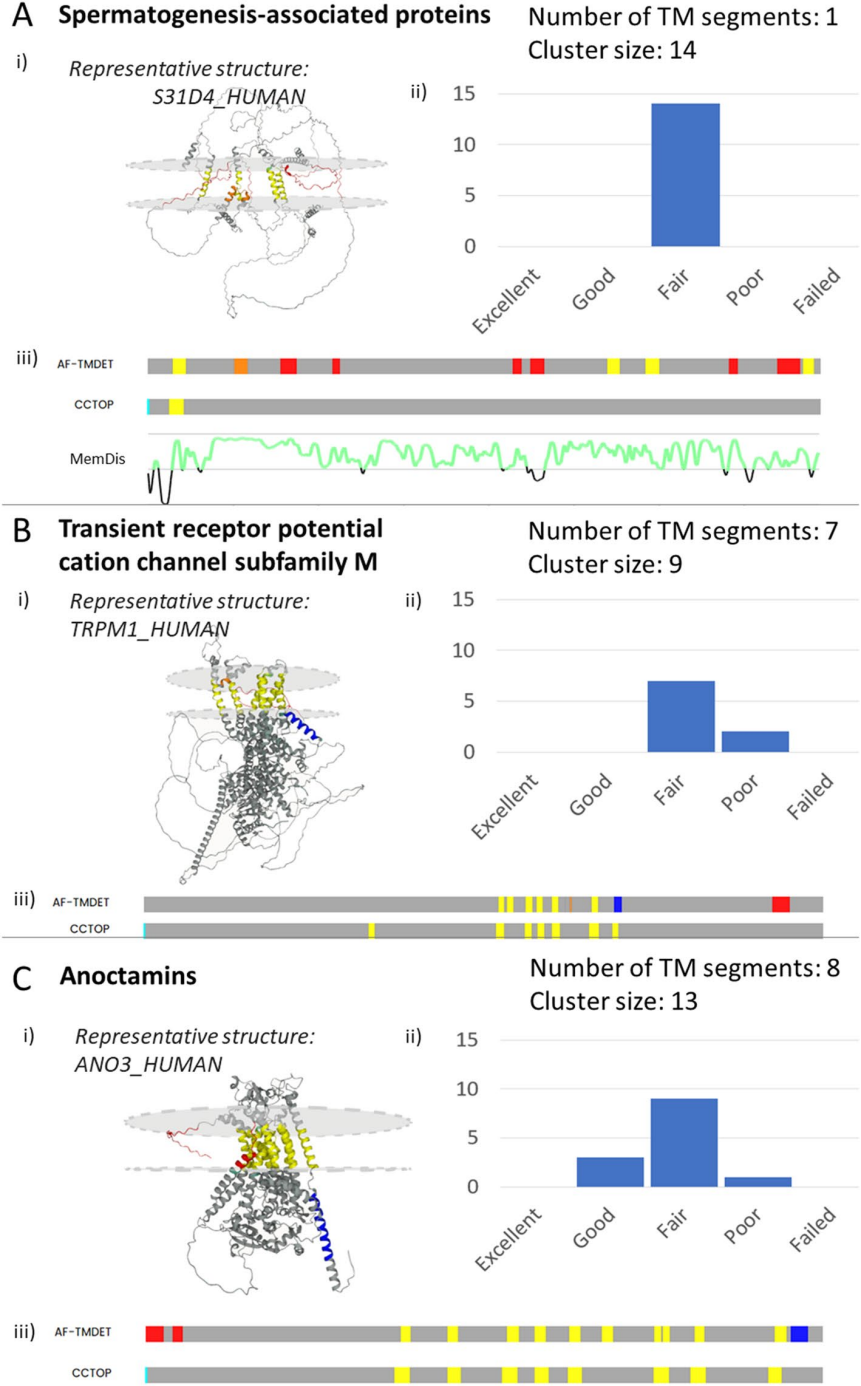
Problematic structures: Examples of badly modeled structures from populated clusters, with a (**i**) representative structure, (**ii**) topography comparison and (**iii**) TMAlphaFold quality level distribution. Top: Spermatogenesis associated proteins (disordered regions are also highlighted using MemDis prediction (green is disordered). Middle: Cation channels. Bottom: Anoctamins.

We found several protein groups where AF2 has limited accuracy on all members. Spermatogenesis-associated proteins are expressed in spermatocytes and the retina, and some of its members are associated with retinal disease (Fig. 5A). HTP predicts them as bitopic membrane proteins, but the reliability of the predictions is always below 85%. Using the AlphaFold2 structures, TMDET^30^ detects 1–5 TM segments with several failed tests, therefore the qualities of all structures are below “fair”. Inspection of the structures shows that some helical segments are present, but the low pLDDT segments connecting them often cross the membrane and place the helical segments randomly. Low pLDDT values indicate flexible disordered regions, and in this case, MemDis^31^ confirms that these proteins are highly disordered. We also found a group of cation channels with 7 TM helices, but all structures are of Poor or Fair quality (Fig. 5B). Several TM segments are folded outside the lipid bilayer, and there are also non-TM regions that are incorrectly predicted to be in the lipid bilayer. In this case, numerous electron-microscopy structures are available that can be used as a template (e.g. PDB: 6MIZ). Anoctamins are anion channels that also seemed to be a challenging task for AF2 (Fig. 5C). They have 8 TM segments, but AF2 tries to make the protein more compact by folding additional helical regions into the lipid bilayer.

**Figure 6:**
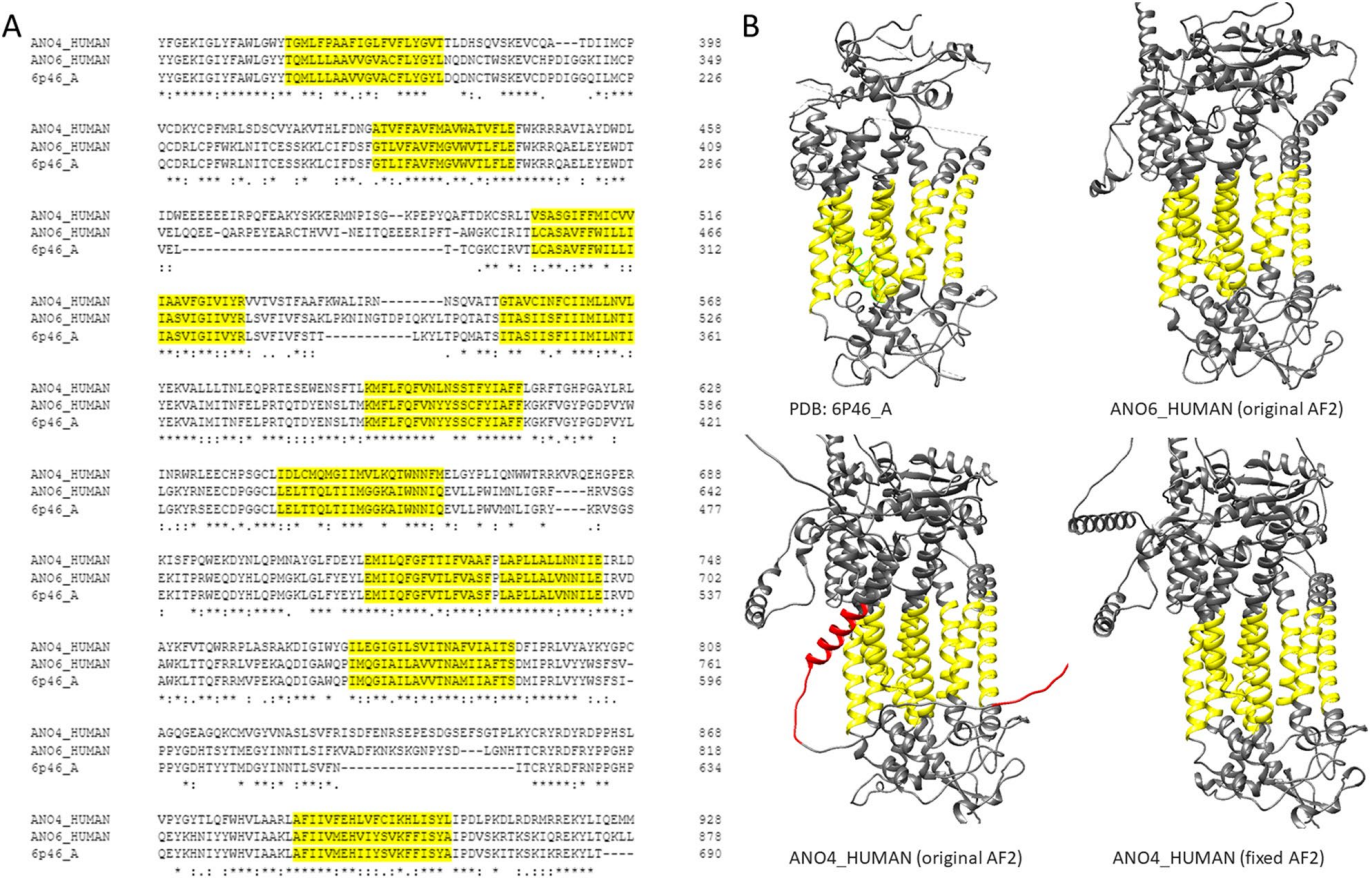
Fixing problematic structures: (**A**) Multiple sequence alignment of human Anoctamin-4, Anoctamin-6 and PDB:6P46_A. (**B**) Top: Electron-microscopy and AF2 predicted structure of Anoctamin-6, Bottom: AF2 predicted structure of Anoctamin-4; AF2 predicted structure of Anoctamin-4 using Anoctamin-6 AF2 structure as a template. Yellow regions show membrane regions as defined by TMDET. Red regions are non-TM regions placed in the plane of the lipid bilayer.

To improve the quality of predictions, one can rely on existing PDB structures, or even on homologous AF2 predictions if their quality is high enough. To demonstrate this, we chose anoctamin-4, which has a helix and a flexible region that violates the lipid bilayer. Multiple sequence alignment to the solved anoctamin-6 structure shows that the TM regions as defined in the PDBTM database are highly conserved (Fig. 6A). Using ColabFold^32^ allows users to select template mode and use one of the electron microscopy structures or the AF2 predicted anoctamin-6 as a template. Using the latter one we successfully amended the erroneous anoctamin-4 membrane domain (Fig. 6B). Older modeling or threading methods, such as SwissModel^33^ or Phyre2^34^ should also not be forgotten. AF2 is trending and has a remarkable performance, but it is also a good practice to use several prediction methods and compare their performance, especially when the AF2 provided structure’s quality is suspicious. TMAlphaFold can be used to assess the quality of AF2 predicted TMP structures to pinpoint problematic proteins.

## Methods

### Performance of AlphaFold on the human transmembrane proteome

First, we prepared a dataset of human proteins for which reasonably high quality structural information was available from PDBTM^3^ structures or topology predictions (reliability > 95% in HTP^1^). Topology predictions include the cytosolic/extracellular localization of segments between TM regions, however in this work we relied only on the location of membrane segments (topography). We used BLAST^21^ with e-value 10^–5^, 40% sequence identity to collect homologous structures for proteins deposited into the HTP database. We only accepted hits that covered all TM segments (3D_set). We extended this set with proteins that have high reliability (> 95%) topography prediction in the HTP (topography_set). This data set was then filtered for redundancy using CD-HIT^35^, by decrementing identity (90, 70, 50, 40) and word length (5,4,3,2). The list of collected proteins can be found in Supplementary Table 1.

We used TM-align^13^ to compare predicted and experimentally solved structures (Supplementary Table 2).

Next, we compared the predicted topography from HTP and the projected topography from AF2 structures, as the membrane bilayer was defined by TMAlphaFold^12^. Correct prediction means that all TM segments overlap by at least 5 residues. The results of the TMAlphaFold filter are also visible here (Supplementary Table 3).

### Coverage of structure on Human Transmembrane Proteins

We downloaded the full UniProt sequence of the PDBTM alpha-helical structures using SIFTS. Using these sequences we searched for homologous hits from HTP using HHBlits^14^, where all PDBTM membrane regions overlap with the HTP sequence in the alignment, with a maximum of 5 gaps allowed—however, as some topography predictions may not be accurate, the predicted transmembrane segments from the HTP were not considered (Supplementary Table 4).

TMAlphaFold quality levels (Supplementary Table 5) and the mapped structures were used to generate Fig. 2A (data: Supplementary Table 6).

### Searching for homologous proteins

We used the UniProt^18^, CATH^17^, Membranome^19^ and the Transporter Classification Database^20^ to search for protein families (Supplementary Table 7). We manually processed the results to construct a dataset, using information from the above listed databases, and in addition from UniProt functional annotations, HTP topographies, PDBTM structures, TMAlphaFold and multiple sequence alignments. We discarded proteins with little or no annotation available, or merged groups where two databases had overlapping results (homology_set, Supplementary Table 8)^18^.

We performed several homology search strategies: BLAST, HHBlits, and Foldseek^29^ (full AF2 structures and only residues in and around the bilayer detected by TMAlphaFold). We also performed the same search but only accepted the result if the topography of the query and the hit protein matched (i.e. all TM regions overlapped by at least 5 residues).

We also developed a simple Neural Network with 1 hidden layer (8 neurons) that takes as input the results of all homology search algorithms (HHBlits result; HHBlits result with overlapping TM regions; BLAST result; BLAST result with overlapping TM regions; Foldseek result; Foldseek result with overlapping TM regions; Foldseek_mem result; Foldseek_mem result with overlapping TM regions), together with TmAlphaFold2 and HTP features of protein pairs (TmAlphaFold number of TM segments; TmAlphaFold quality; HTP number of TM segments; HTP reliability). We trained these networks for 10 epochs. To avoid overfitting, we created several training and test sets—always using one protein family to test and the rest of the protein families to train/validation (Supplementary Table 9, Supplementary Fig. 2). The results of the individual predictors on the train/validation/test sets are in Supplementary Table 10. For the final prediction, we aggregated the results from individual predictors. To further ensure NNSearch is not overfitting, we also performed a bootstrap evaluation on it (and on all other methods), by holding back 20% of the data 100 times (Supplementary Table 11).

We also used several clustering techniques: as protein sequence identity and structural similarity varied greatly between families, we used DBScan^22^ to cluster the results (as it is a density-based clustering method) using the similarity matrix provided by the search tools. We iterated through different epsilon values and selected the one with the highest Adjusted RandIndex and Adjusted Mutual Information. We also used Foldseek clustering on full and partial structures (with residues in and around the bilayer), using thresholds of 0.2, 0.4, 0.6, 0.8. The selection criteria were the same as for DBScan (Supplementary Tables 10–13). Cluster results for the homology_ set are shown in Supplementary Table 14.

### Searching for groups with low quality AlphaFold structures

We used the developed Neural Network (NNSearch) to define protein clusters (Supplementary Table 15). Clusters with a mean quality of less than 3.5 (transforming Failed-Excellent to 1–5 scores, Supplementary Table 16) and more than 10 proteins were further analyzed in the Discussion (Supplementary Table 17). GeneOntology analyses and enrichment calculations were performed using GOrilla^36^, by selecting genes with Fair or lower quality and using the full human TM proteome as a background. Highly significant terms (*P*-value <  = 10^–10^) were selected and categorized according to their depth in the ontology (Supplementary Table 18).

### Supplementary Information

Supplementary Figures.

Supplementary Tables.

## Data Availability

All data generated or analysed during this study are included in this published article and its supplementary information files.
