# Supplementary material for: How AlphaFold2 shaped the structural coverage of the human transmembrane proteome
Source: Sci Rep. 2023 Nov 20;13:20283. doi: 10.1038/s41598-023-47204-7 (PMC10662385; doi:10.1038/s41598-023-47204-7)
Supplement: Supplementary file 1 — Supplementary Figures. [file 41598_2023_47204_MOESM1_ESM.pdf]

Supplementary Material for How AlphaFold shaped the structural coverage of the human transmembrane proteome

Márton A. Jambrich[1], Gabor E. Tusnady[1,2,*], Laszlo Dobson[1,2,3]
[1]Protein Bioinformatics Research Group, Institute of Enzymology, Research Centre for Natural Sciences, Magyar Tudósok körútja 2, H-1117 Budapest, Hungary
[2]Department of Bioinformatics, Semmelweis University, Tűzoltó u. 7, H-1094 Budapest, Hungary
[3]Structural and Computational Biology Unit, European Molecular Biology Laboratory, Meyerhofstraße 1, 69117 Heidelberg, Germany
*To whom correspondence should be addressed. Tel: +36 1 382 6709; Fax: +36 1 382 6295; Email: tusnady.gabor@ttk.hu

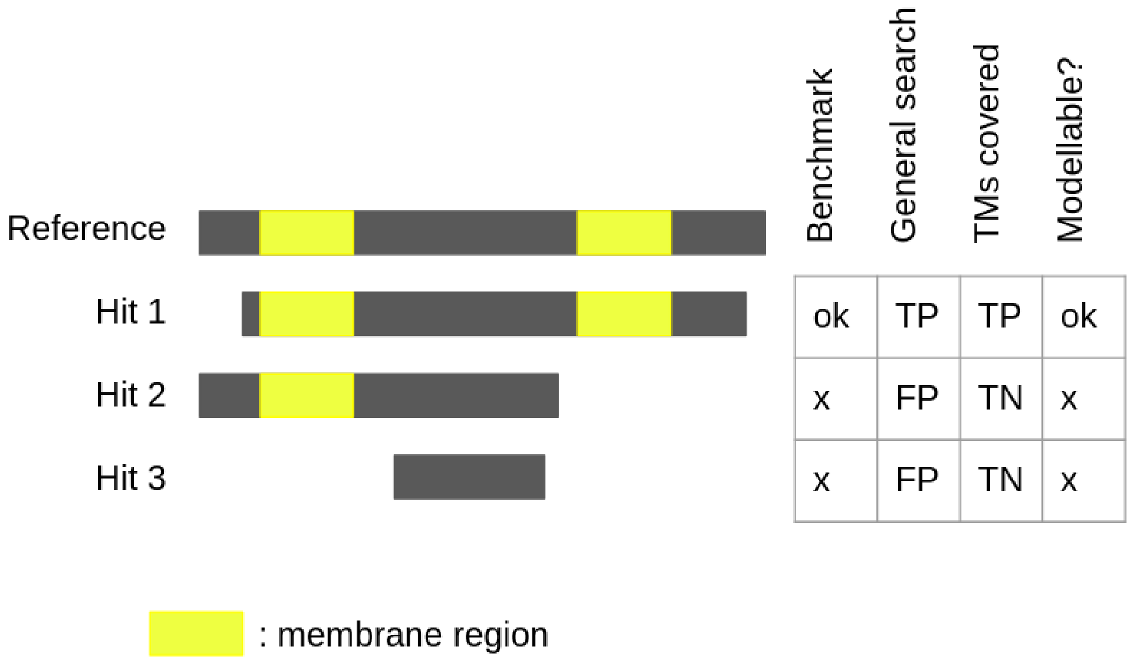

Supplementary Figure 1: Logic of how homology searching results were handled. In the benchmark set we only accepted pairs of proteins where all TM regions overlapped. In general search, all kinds of hits were accepted. In the next search we required TMs to be covered, which eliminates Hit2/Hit3. If someone would like to model the structure of the TM domain, only hit 1 can be used as a reference (for homology modeling).

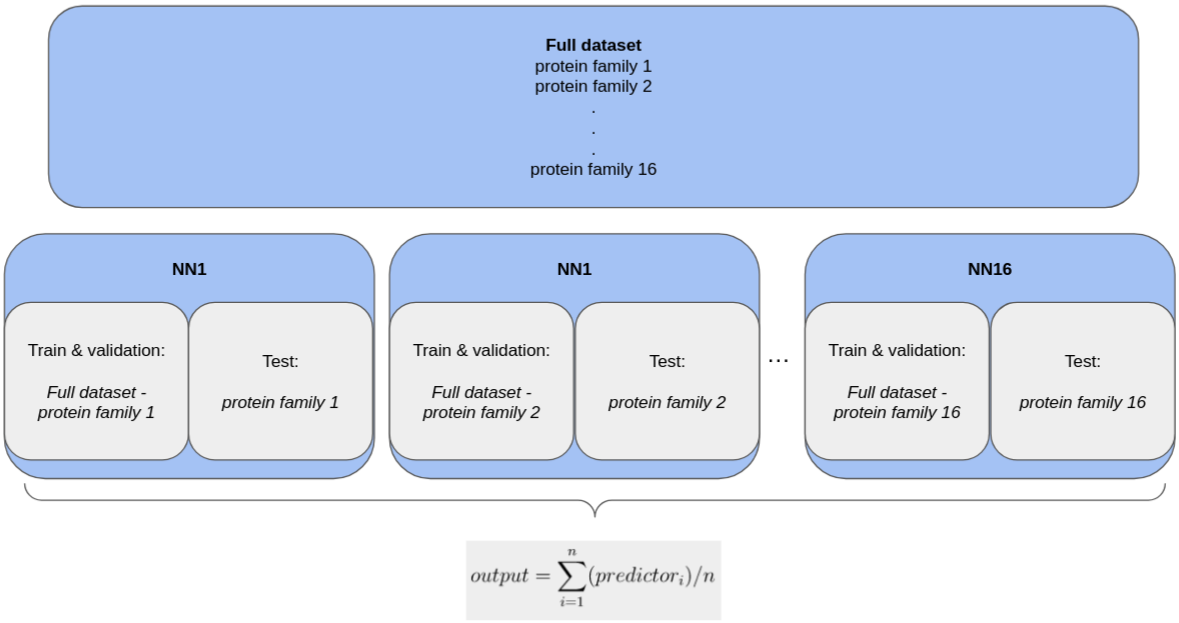

Supplementary Figure 2: Schematic graph of data processing in NNSearch
